# Investigation of c-Fos/c-Jun Signaling Pathways in Periostracum Cicadae’s Inhibition of EMT in Gastric Tissue

**DOI:** 10.3390/ph18040537

**Published:** 2025-04-07

**Authors:** Hua Liang, Xiaofei Jin, Tongtong He, Xiaohong Zhou, Zhenyi Liu, Weijuan Gao

**Affiliations:** Hebei Key Laboratory of Chinese Medicine Research on Cardio-Cerebrovascular Disease, Hebei University of Chinese Medicine, No. 3 Xingyuan Road, Luquan District, Shijiazhuang 050200, China; 15176870735@163.com (H.L.); jxf1655@163.com (X.J.); halona0211@163.com (T.H.); zzxxhh2007@126.com (X.Z.)

**Keywords:** Periostracum Cicadae, chronic atrophic gastritis, epithelial–mesenchymal transition, c-Fos/c-Jun signaling pathway

## Abstract

**Background/Objectives**: Periostracum Cicadae (PC) is commonly used to treat chronic atrophic gastritis (CAG), but its underlying mechanisms are unclear. We investigated the therapeutic effects, active ingredients and molecular mechanisms of PC on CAG. **Methods**: We analyzed the components in the serum extract of PC by UHPLC-Q-Orbitrap-MS/MS. Then, we used rat and cell models to assess the impact of PC on CAG and employed network pharmacology and bioinformatics to predict key targets and active ingredients. Finally, we confirmed hub targets through experiments and molecular docking. **Results**: A total of 22 components were identified in the PC extract-containing serum using UHPLC-Q-Orbitrap MS/MS. Network pharmacology combined with molecular docking revealed that the protective effect was primarily mediated by three compounds: (Z)-akuammidine, chicoric acid, and columbianadin. And we revealed that c-Fos/c-Jun signaling pathways were crucial in therapy. PC extract-containing serum inhibited the vitality, migration, invasion, and multiplication of MC cells (model cells for CAG), induced apoptosis, and caused G0/G1 phase cell cycle arrest. The expression level of tumor necrosis factor-alpha (TNF-α), interleukin-6 (IL-6), interleukin-1 beta (IL-1β) and gastrin 17 (G17) in the serum of CAG rats increased, while the expression level of pepsinogen I (PG I) and pepsinogen II (PG II) decreased. After 12 weeks of PC administration, these conditions were significantly improved. PC not only reduced the levels of antigen KI-67 (Ki67) and tumor protein p53 (P53) but also enhanced SRY-box Transcription Factor (SOX2). Simultaneously, PC down-regulated the expression of N-cadherin and Vimentin while up-regulating that of E-cadherin. **Conclusions**: PC inhibited epithelial–mesenchymal transition (EMT) via the c-Fos/c-Jun signaling pathway, thereby providing therapeutic benefits for CAG. Our study elucidates the mechanisms and material basis of PC in treating CAG, providing experimental evidence to support its clinical application.

## 1. Introduction

Chronic atrophic gastritis (CAG) is a digestive disease whose main features include atrophy of the gastric mucosa, exposed blood vessels, and the formation of mucosal nodules [1]. CAG is multifactorial and has been associated with *Helicobacter pylori* (Hp) infection, bile reflux, and dietary habits [2]. CAG is a condition that precedes gastric cancer (GC), so its early diagnosis and treatment are essential to prevent the development of malignant tumors [3]. The fundamental therapeutic approach for CAG involves eradicating Hp using antibiotics. In conventional treatment, gastroprotective agents are also advisable [4]. However, in advanced CAG, Hp eradication therapy may inadequately halt gastric gland atrophy progression, while the long-term benefits and safety profiles of certain gastroprotective agents remain understudied [4,5].

The epithelial–mesenchymal transition (EMT) is a process in which tumor cells transdifferentiate into a mesenchymal phenotype, acquiring enhanced migratory and invasive capacities, and it is closely associated with the inflammation-to-cancer transition [6,7]. Gastric mucosal inflammatory response triggers the secretion of pro-inflammatory cytokines, which induce EMT [8]. The study indicated that Traditional Chinese Medicine (TCM) compound prescriptions treat CAG by inhibiting JAK2/STAT3-mediated EMT, curbing gastric inflammation–cancer transition [9].

TCM has demonstrated remarkable effectiveness in the treatment of CAG [10,11]. Research findings have indicated that specific TCM prescriptions like Shen-Ling-Bai-Zhu-San possess potential therapeutic mechanisms and clinical proof in the treatment of chronic gastritis [12]. Moreover, Modified Sijunzi Decoction (MSD) has been proven to relieve CAG symptoms and ameliorate pathological alterations [13]. These herbs may play a role in the treatment of CAG by modulating the immune system and inflammatory response [14,15].

Periostracum Cicadae (PC) is the skin shell shed by the black cicada (*Cryptotympana pustulata* Fabricius), an insect of the Cicadidae family, when it molts. PC has been extensively applied in TCM for diverse ailments [16,17]. PC was first documented in the *Ming-I-Pieh-Lu* (an important medical text from ancient China) for treating pediatric convulsions and fever [18]. PC has the efficacy of “clearing heat and removing toxins”. Contemporary pharmacological investigations substantiate its anti-inflammatory capacity, which is mediated through multiple pathways. PC regulates key signaling pathways implicated in inflammatory responses and tumor progression through many mechanisms: the suppression of pro-inflammatory cytokine production and disruption of oncogenic signaling cascades with a concomitant modulation of apoptosis-associated proteins [19,20]. Notably, N-acetyldopamine derivatives, a component of PC, can inhibit NF-κB and MAPK pathways and reduce the release of inflammatory mediators, thereby alleviating colonic mucosal inflammation [21]. For chronic diseases of the stomach, PC significantly alleviated the symptoms of gastric pain, distension, belching, and noisiness. However, the molecular targets and bioactive components require systematic exploration.

In recent years, the integration of computational simulation techniques and experimental investigations has demonstrated significant potential in the field of Chinese medicine research [22]. Serum medicinal chemistry and network pharmacology have emerged as advanced methodologies for elucidating the mechanisms of action of herbal medicines [23]. In this study, we extracted PC extract-containing serum from rats. Furthermore, ultra-performance liquid chromatography-high resolution mass spectrometry (UPLC-HRMS) was used to precisely identify the compounds present in the PC extract-containing serum. By integrating data from disease- and drug-related databases, we screened key compounds in PC and predicted their potential primary targets. Subsequently, we conducted functional enrichment analyses, including Gene Ontology (GO) and Kyoto Encyclopedia of Genes and Genomes (KEGG) analyses, along with bioinformatics analyses, to thoroughly elucidate the principal mechanisms. In addition, we applied molecular docking techniques to validate the ligand–target interactions between the compounds and key predicted targets. Finally, we validated the efficacy of the PC and its key targets through in vivo and in vitro experiments, which further ensured the reliability and accuracy of the study results.

## 2. Results

### 2.1. UHPLC-Q-Orbitrap-MS/MS Analysis of the PC Extract-Containing Serum

**Figure 1:**
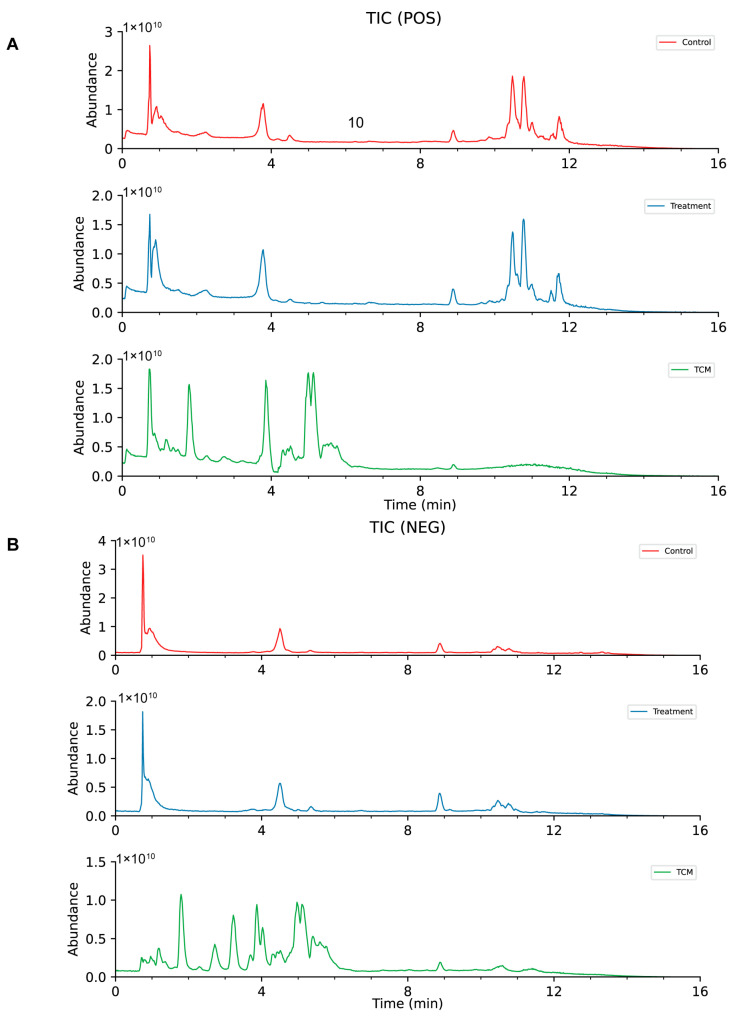
(**A**). Total ion chromatograms (TIC) acquired in the positive electrospray ionization (ESI^+^) mode (**B**). TIC obtained in negative ionization (ESI^−^) mode.

The primary constituents of the PC extract-containing serum were analyzed using UHPLC-Q-Orbitrap-MS/MS (Figure 1), and a total of 22 identifiable compounds were identified. The analysis included flavonoids (e.g., (+)-afzelechin MOL01), alkaloids ((Z)-akuammidine, MOL02), and phenolic acids (chicoric acid, MOL10). High mass accuracy (ΔMass < ±5 ppm) was observed, which was exemplified by MOL01 (0.78 ppm) and MOL07 (0.04 ppm). Retention times (RTs) spanned 2.47–13.19 min with hydrophobic compounds like MOL20 (RT = 11.92 min) eluting later. Adducts varied by ionization mode: [M-H]—(NEG mode, e.g., MOL04) and [M+H] + (POS mode, e.g., MOL08) were prominent (Table 1). The method’s efficacy was affirmed by robust chromatographic separation and high-resolution MS data, demonstrating its suitability for comprehensive serum profiling.

### 2.2. Hub Targets for PC Therapy of CAG

**Figure 2:**
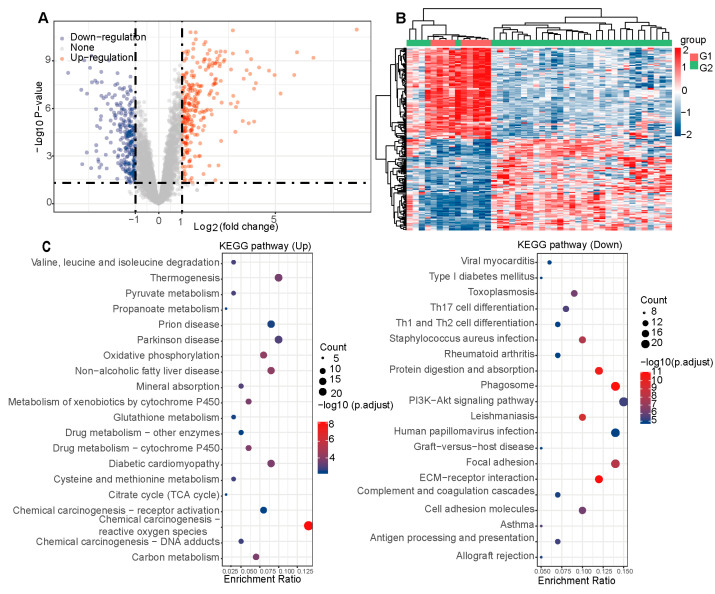
(**A**) The volcano plots of gene expression between normal and GC; (**B**) the clustered heat maps of samples between normal and GC; (**C**) the KEGG enrichment analysis in normal and GC.

A total of 525 differential genes were found between normal and GC tissues, of which 256 genes were down-regulated and 269 were up-regulated. Visualization through volcano plots and clustered heat maps clearly demonstrated the distinct distribution of gene expression levels between normal and GC (Supplementary Table S1, Figure 2A,B). Subsequently, we analyzed the Differentially Expressed Genes (DEGs) using KEGG. The KEGG enrichment analysis indicated that the PI3K-Akt signaling pathway may be involved in the regulation of GC (Figure 2C).

**Figure 3:**
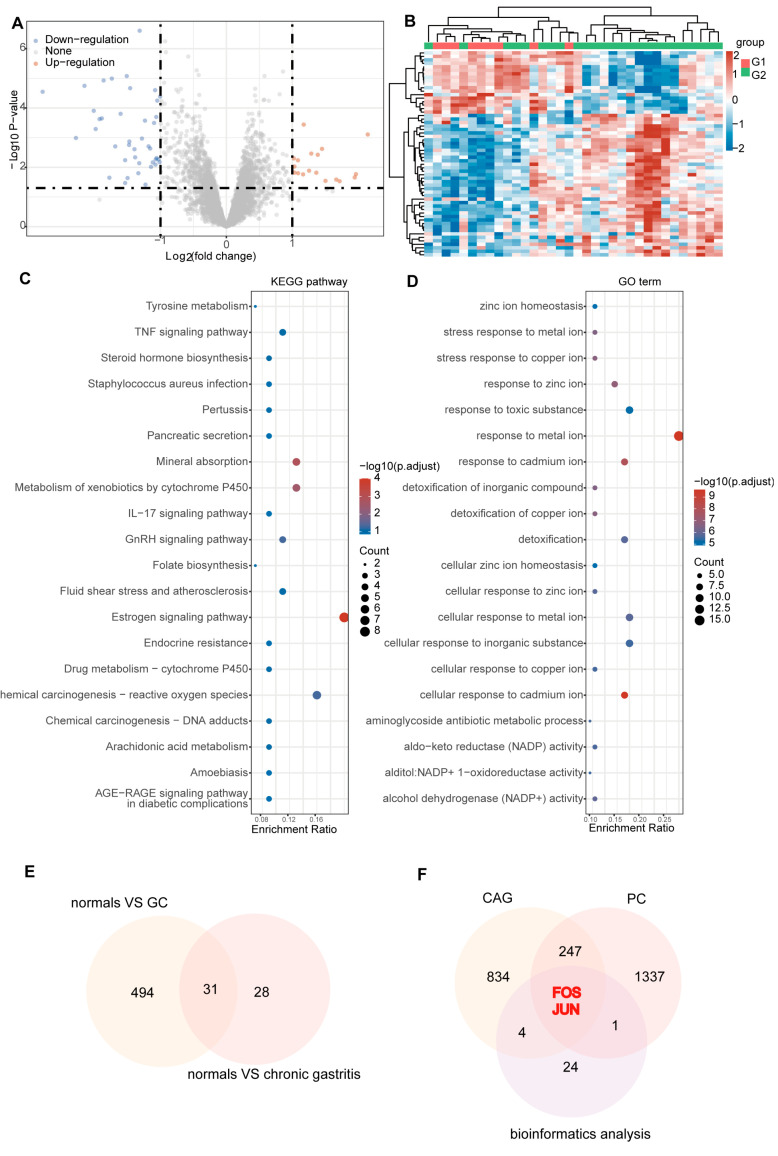
(**A**) The volcano plots of gene expression between normal and chronic gastritis; (**B**) the clustered heat maps of samples between normal and chronic gastritis; (**C**) the KEGG enrichment analysis in normal and gastritis; (**D**) the GO enrichment analysis in normal and chronic gastritis; (**E**) Venn diagram of targets intersection of bioinformatics analysis; (**F**) Venn diagram of targets intersection of CAG-PC-bioinformatics analysis. GO: Gene Ontology; KEGG: Kyoto Encyclopedia of Genes and Genomes.

We identified 59 DEGs in normal and chronic gastritis tissues, which included 18 up-regulated and 41 down-regulated genes. The volcano plots and clustered heat maps clearly demonstrated the distinct distribution of gene expression (Supplementary Table S2, Figure 3A,B). The KEGG enrichment analysis indicated that the TNF signaling pathway may be involved in the regulation of chronic gastritis tissues, while the GO enrichment pathways were mainly some ionic pathways (Figure 3C,D).

A total of 31 intersecting genes were found by intersecting genes that differed among normal, chronic gastritis and GC (Figure 3E). Overall, 1586 targets related to PC were predicted, while 1087 targets of CAG were collected (Supplementary Figure S1 and Figure 3F). Ultimately, a combined network pharmacology and bioinformatics analysis identified FOS and JUN as the hub genes for the PC amelioration of CAG symptoms. Subsequent experiments focused on their protein products, c-Fos and c-Jun.

### 2.3. MC Cells Model Establishment and Validation

**Figure 4:**
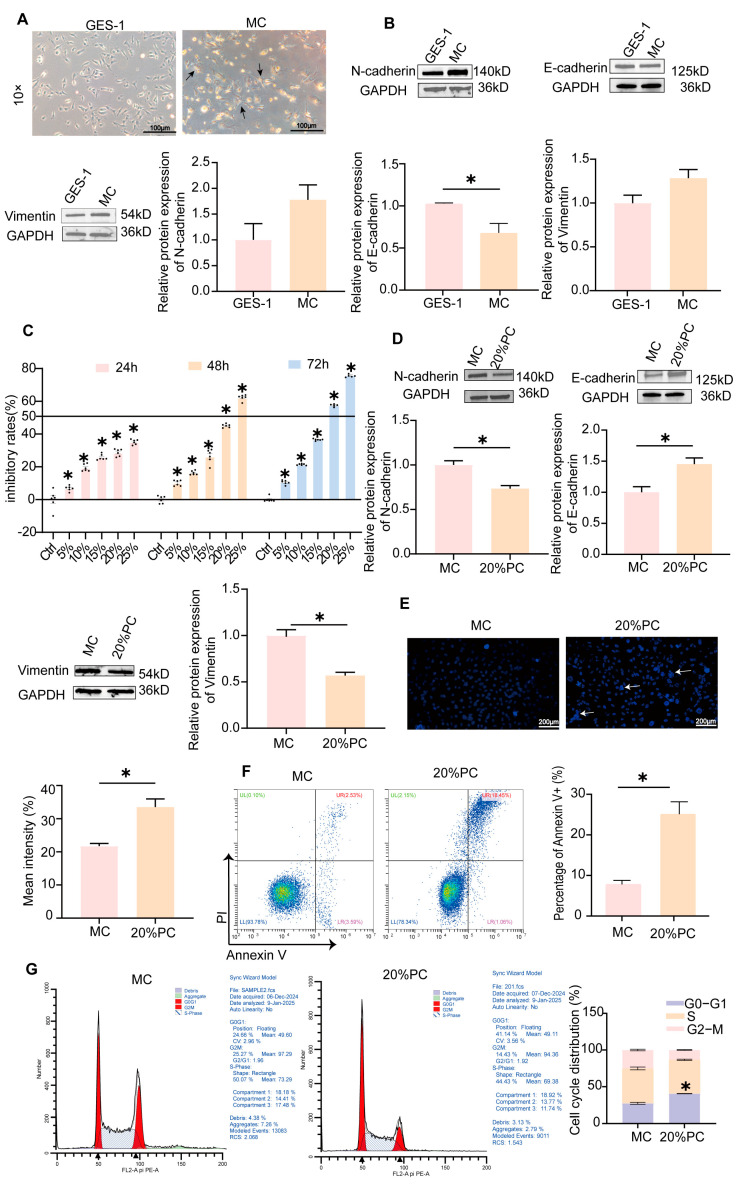
(**A**) Representative images of cells; Arrows indicate irregular morphology and characteristic “island-like” clonal growth patterns (see Section 2.3 for details). (**B**) expression of EMT-related proteins (N-cadherin, E-cadherin, Vimentin) in GES-1 cells and MC cells was determined by Western blot. Protein levels were quantified using ImageJ 1.54f; (**C**) effect of PC on MC cell viability at 5–25% serum concentration; (**D**) expression of EMT-related proteins (N-cadherin, E-cadherin, Vimentin) in MC cells and cells treated with 20% PC extract-containing serum was analyzed by Western blot. Protein levels were quantified using ImageJ 1.54f; (**E**) Hoechst 33342 staining of MC cells and 20% PC-treated cells observed by fluorescence microscopy. Fluorescence intensity was quantified and presented as bar graphs; Arrows represent MC cells treated with 20% PC extract-containing serum displayed characteristic apoptotic features (as described in Section 2.3). (**F**) apoptosis of MC cells treated with 20% PC was analyzed by Annexin V/PI staining. Apoptotic rates are shown in bar graphs; (**G**) cell cycle distribution of MC cells treated with PC was analyzed by PI staining. DNA content was measured to assess G0/G1, S, and G2/M phases. All data are expressed as mean ± SEM. * *p* < 0.05.

We treated GES-1 cells with N-methyl-N′-nitro-N-nitrosoguanidine (MNNG) to establish a cellular model of CAG, which was designated as MC cells. The MC cells displayed irregular morphology and characteristic “island-like” clonal growth patterns; this differed from the orderly arrangement and spindle-shaped morphology of the GES-1 cells (Figure 4A). EMT, a hallmark of MC cells, involves the decrease in cell adhesion, the increase in epithelial markers and the decrease in mesenchymal markers [24,25]. A comparison with GES-1 cells revealed that MC cells exhibited a significant reduction in E-cadherin expression and an elevated expression of N-cadherin and Vimentin (*p* < 0.05, Figure 4B). These results validated the successful generation of MC cells, providing a foundation for further cellular experiments in subsequent studies.

CCK-8 assay demonstrated that PC extract-containing serum inhibited MC cell proliferation in a concentration-dependent manner. In particular, the inhibitory rate of 20%PC extract-containing serum on MC cells for 48 h was 50%, so we chose this condition for follow-up experiments (*p* < 0.05, Figure 4C). Moreover, the 20% extract-containing serum of PC exerted an inhibitory influence on the expression of N-cadherin and Vimentin in MC cells significantly while enhancing the expression of E-cadherin protein significantly (*p* < 0.05, Figure 4D).

The detection of MC cell apoptosis is crucial for understanding the mechanisms underlying CAG. Hoechst 33342 staining revealed that MC cells treated with 20% PC extract-containing serum displayed characteristic apoptotic features, including nuclear condensation, fragmentation, and apoptotic bodies, with significantly enhanced fluorescence intensity compared to untreated controls (*p* < 0.05, Figure 4E). Flow cytometry analysis demonstrated that 20% PC extract-containing serum significantly induced apoptosis in MC cells (*p* < 0.05, Figure 4F). Beyond apoptosis, PC significantly influenced the cell cycle: the percentage of cells in the G0/G1 phase escalated from 24.66% to 41.14%, whereas the proportion in the G2/M phase diminished from 25.27% to 14.43% (*p* < 0.05, Figure 4G).

**Figure 5:**
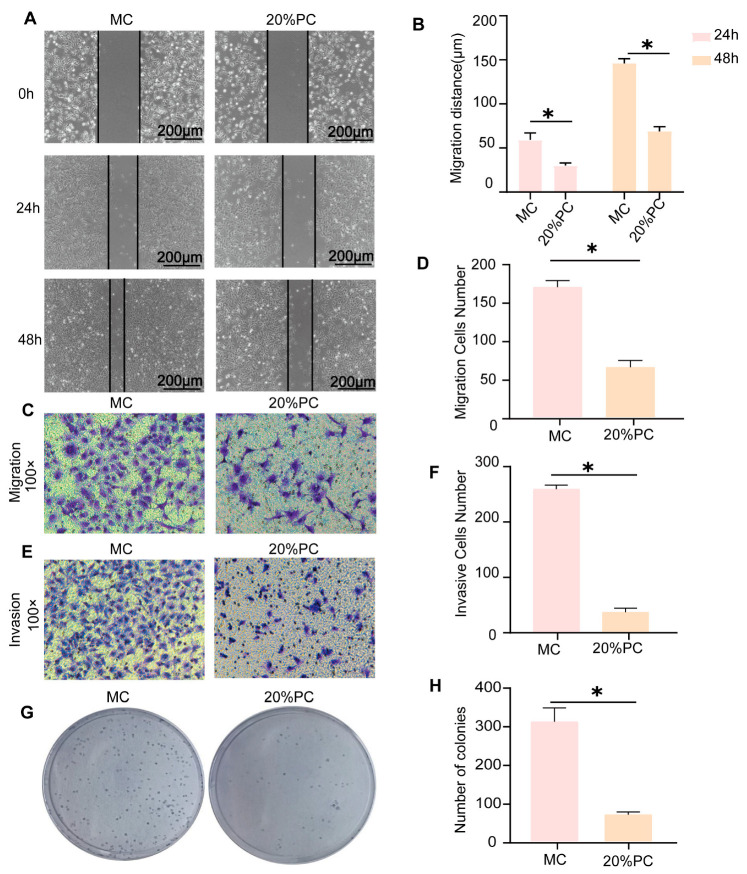
PC extract-containing serum suppresses the migration, invasion, proliferation and clonogenicity of MC cells. (**A**,**B**) scratch assay assessing PC’s effect on MC cell migration. Wound closure was monitored at 24 h and 48 h using inverted microscopy. Distance of migration is shown in bar graphs. (**C**,**D**) transwell migration assay evaluating PC’s effect on MC cell motility. Magnified views: 100× in (**C**). (**E**,**F**) matrigel-coated transwell invasion assay assessing PC’s inhibitory effect. The number of cells passing through the chamber is shown in bar graphs. Magnified views: 100× in (**E**). (**G**,**H**) colony formation assay demonstrating PC’s suppression of MC cell clonogenicity. Colonies were counted after crystal violet staining and statistically analyzed. All data are expressed as mean ± SEM. * *p* < 0.05.

The scratch test demonstrated that PC significantly inhibited the migratory capacity of MC cells, the migration distance was significantly reduced at 24 and 48 h *(p* < 0.05, Figure 5A,B). Furthermore, transwell migration and invasion assays demonstrated that PC significantly inhibited the migratory and invasive abilities of MC cells (*p* < 0.05, Figure 5C–F). Cell cloning experiments further substantiated that PC suppresses the proliferation of MC cells (*p* < 0.05, Figure 5G,H). Collectively, these findings suggest a strong correlation between the effects of PC on MC cell proliferation, migration, and invasion and the EMT process.

### 2.4. PC Exhibited Therapeutic Effects in CAG Rats

**Figure 6:**
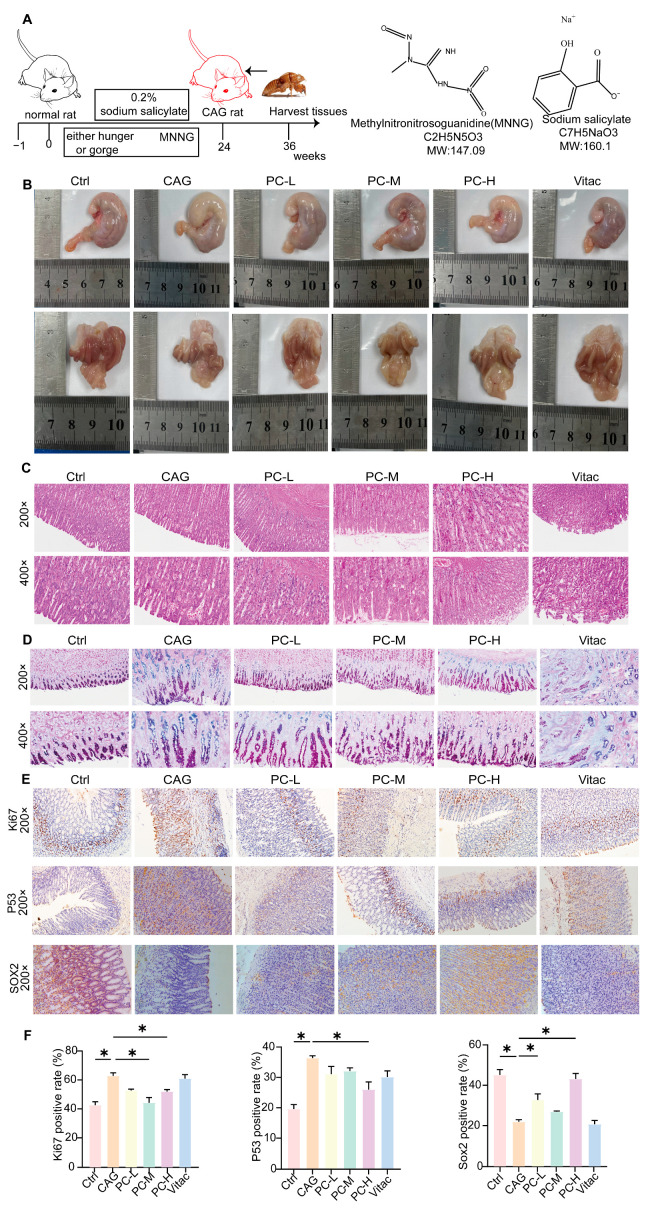
(**A**) CAG rats modeling cycle and chemical structure of the drug used for modeling. (**B**) Representative morphology of stomach image of rats; (**C**) Typical representative sections HE of gastric histopathology in rats; Magnified views: 200×; 400×. (**D**) Assessment of intestinal chemotaxis using AB-PAS; Magnified views: 200×; 400×. (**E**) Representative IHC images of Ki67, P53 and SOX2 are shown; Magnified views: 200×. (**F**) The statistical chart of immunohistochemistry staining. Ctrl, control; PC-L, PC low-dose group; PC-M, PC middle-dose group; PC-H, PC high-dose group. All data represent the mean ± SEM. * *p* < 0.05.

The CAG animal model was established by employing MNNG as the primary carcinogen, in conjunction with sodium salicylate—induced gastric mucosal injury, and was further supplemented by inducing hunger—satiety abnormality. The experimental cohort received MNNG (180 μg/mL) via drinking water ad libitum, which was coupled with cyclical feeding patterns (2-day ad libitum feeding alternating with 24 h fasting). Rats received intragastric administration of 2% sodium salicylate after a fasting period. (1 mL per 100 g body weight). This integrated protocol was maintained cyclically for 24 weeks. Then, PC was administered via oral gavage at varying doses for 12 weeks (Figure 6A). In comparison to the control group, the CAG group displayed gastric mucosal congestion and a reduction in wrinkles. This condition showed improvement following the administration of various concentrations of PC and Vitacoenzyme tablet (Vitac) (Figure 6B). Histopathological analysis revealed that the rats of CAG displayed thinning of the gastric mucosal layer, a decrease in the number of inherent glands, gland atrophy, and a scanty and disorderly arrangement of glands. Furthermore, the results showed epithelial cell degeneration, a loss of parietal cells, and the significant infiltration of granulocytes and mononuclear cells in the mucosa and submucosa. Post-treatment, the lesions exhibited varying degrees of recovery (Figure 6C). Alcian Blue–Periodic Acid-Schiff staining (AB-PAS) was performed to assess intestinal metaplasia severity. In normal gastric mucosa, neutral mucins were stained magenta, while acidic mucins in intestinal metaplasia lesions exhibited blue coloration. The CAG group displayed prominent intestinal metaplasia in gastric mucosa, which was significantly attenuated after PC treatment (Figure 6D).

Abnormal cell division induced by over-proliferation increases the risk of developing precancerous lesions [26]. Our study demonstrated a significant overexpression of antigen KI-67 (Ki67) and tumor protein p53 (P53) in CAG rats. However, following treatment, PC markedly reduced the expression levels of Ki67 and P53 (*p* < 0.05, Figure 6E,F). Chronic inflammation not only leads to mutations in the P53 gene but also suppresses SRY-box Transcription Factor (SOX2) expression, thereby elevating the risk of GC development. In CAG rats, SOX2 expression was significantly lower compared to the normal group, whereas varying doses of PC notably enhanced SOX2 expression (*p* < 0.05, Figure 6E,F).

**Figure 7:**
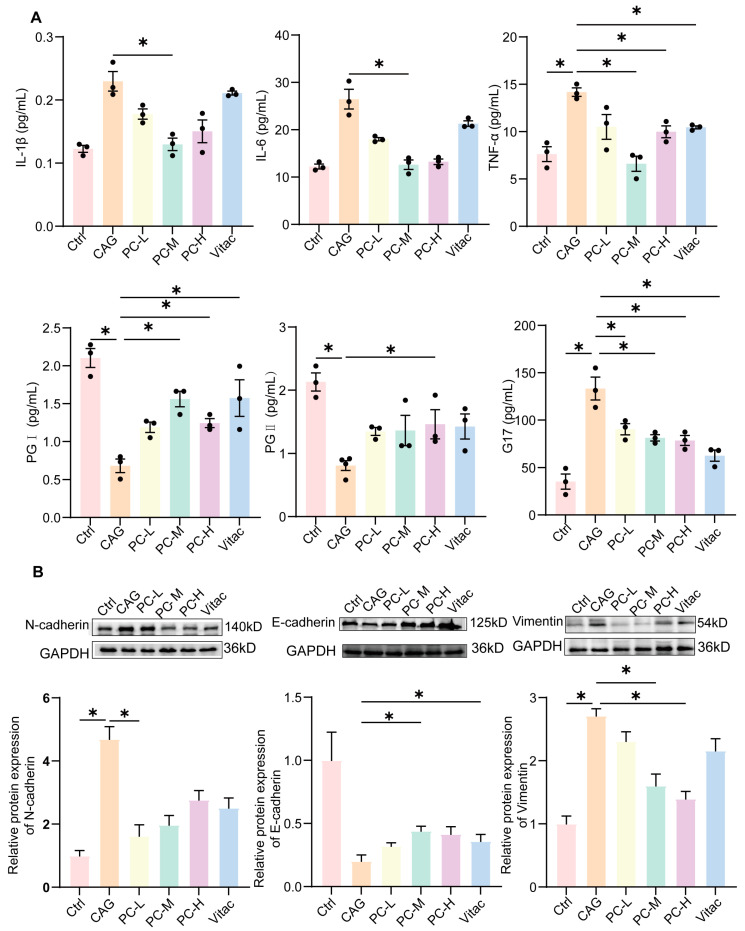
(**A**) Serum levels of IL-1β, IL-6, TNF-α, PG I, PG II, and G17 were measured to evaluate the effect of PC. (**B**) EMT-related protein expression in gastric tissues. Western blot analysis of N-cadherin, E-cadherin, and Vimentin expression. Protein bands were quantified by densitometry using ImageJ 1.54f. All data represent the mean ± SEM. * *p* < 0.05.

The elevated serum levels of tumor necrosis factor-alpha (TNF-α), interleukin-6 (IL-6) and interleukin-1 beta (IL-1β) contribute to gastric mucosal damage and accelerate the transition from CAG to GC. In comparison to the control group, the CAG group demonstrated elevated expression levels of IL-1β, IL-6, and TNF-α. However, this increase was mitigated by the treatment of PC and Vitac (*p* < 0.05, Figure 7A). Furthermore, we investigated the changes in the gastric function markers gastrin 17 (G17), pepsinogen I (PG I) and pepsinogen II (PG II). The serum levels of PG I and PG II were significantly decreased in CAG rats compared to the control group, whereas the levels of G17 were increased. Following a 12-week treatment, the PC group exhibited increased levels of PG I and PG II compared to the CAG group, while the levels of G17 were significantly decreased (*p* < 0.05, Figure 7A).

In addition, the expression of N-cadherin and Vimentin in CAG rats after PC treatment was significantly inhibited, and the expression of E- cadherin was significantly enhanced (*p* < 0.05, Figure 7B).

### 2.5. PC Improves the Symptoms of CAG Through c-Fos/c-Jun

**Figure 8:**
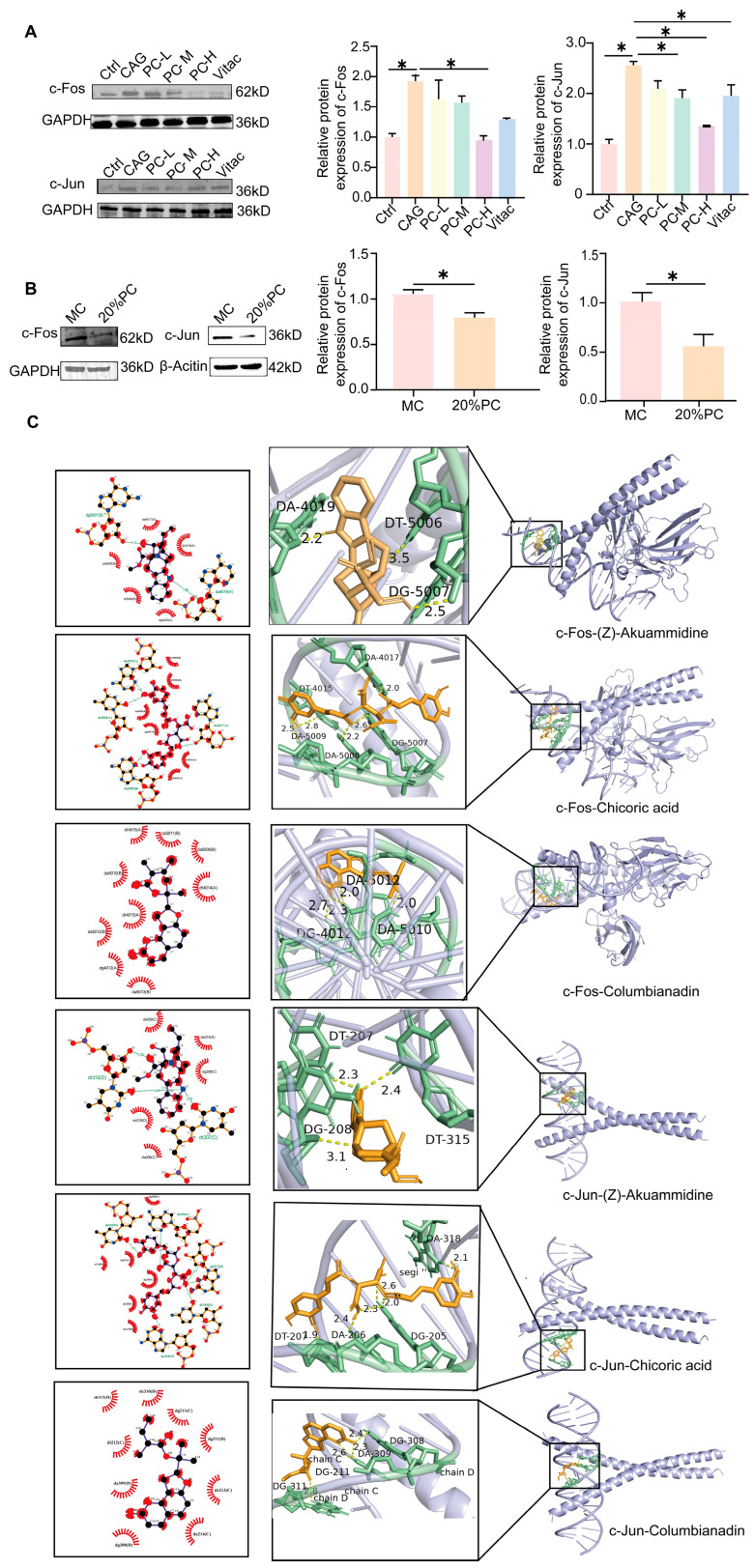
Verification of key targets: (**A**) c-Fos and c-Jun protein levels in gastric tissue of rats were analyzed by Western blot; Protein bands were quantified by densitometry using ImageJ 1.54f. (**B**) c-Fos and c-Jun protein levels in MC cells were analyzed by Western blot; Protein bands were quantified by densitometry using ImageJ 1.54f. All data represent the mean ± SEM. * *p* < 0.05. (**C**) Molecular docking of potential active components of PC with c-Fos/c-Jun.

c-Jun and c-Fos are key transcription factors driving the pathogenesis of precancerous lesions by regulating inflammatory responses, cell proliferation, and apoptosis [27,28]. In CAG rats, the expression levels of c-Fos and c-Jun were significantly elevated. However, treatment with PC effectively restored these levels to normal. Similarly, in MC cells, serum containing PC was observed to reduce the expression of c-Jun and c-Fos (Figure 8A,B).

Using Cytoscape, we constructed a PC-compounds-targets-pathways–CAG interaction network wherein the top five degree-ranked components were identified as potential active constituents of PC against CAG (Supplementary Figure S1G and Table S3). They were (Z)-akuammidine, chicoric acid, columbianadin, gamma-l-glutamyl-l-tyrosine and guaiacin.

Generally, a docking score less than −7.0 k/mol indicates strong binding activity. We show fractions with binding energies below −7.0 k/mol after both c-Fos and c-Jun binding (Supplementary Table S4, Figure 8C). Collectively, we predict that the three compounds may play a key role in the treatment of CAG. They are (Z)-akuammidine, chicoric acid, columbianadin.

## 3. Discussion

CAG is a significant precursor to GC, making timely diagnosis and intervention essential to prevent its progression to malignancy [29]. Recent studies have shown that specific herbal extracts and natural compounds exhibit both therapeutic and preventive effects on CAG, leading to notable improvements in clinical symptoms and gastroscopic pathology [9]. PC, a commonly used Chinese medicine in the clinical treatment of CAG, has demonstrated substantial therapeutic effects. However, its underlying molecular mechanisms remain largely unexplored. To identify the effective constituents of PC, we employed UPLC-Q-Exactive Orbitrap MS/MS technology, which revealed a total of 22 major compounds, including phenolic acids, flavonoids, triterpenes, etc.

Subsequently, through network pharmacology analysis, we identified the c-Fos/c-Jun signaling pathway as a potential mechanism of action, which was classically associated with the EMT process [30]. In EMT, c-Fos/c-Jun is activated by diverse signaling pathways. These two factors bind to specific DNA sequences, thereby promoting cell motility and invasion, further advancing the EMT progression [31].

EMT promotes the malignant progression of CAG to GC by down-regulating E-cadherin, a key epithelial adhesion molecule, thereby disrupting intercellular junctions and cellular cohesion, concomitantly up-regulating N-cadherin to potentiate cellular motility [32,33]. Notably, the observed elevation in Vimentin expression drives cytoskeletal reorganization, enabling dynamic cellular plasticity and augmented migratory competence across heterogeneous tissue matrices [34]. The AP-1 constituent proteins c-Jun and c-Fos serve as master regulators governing the delicate balance between mitotic activity and apoptotic signaling cascades in cellular systems [35]. c-Jun is able to form an AP-1 complex with c-Fos, which accelerates cell proliferation by promoting the expression of cell cycle genes, and it modulates apoptosis via dynamic interactions with P53 [36,37,38]. In chronic inflammatory environments, persistent c-Fos and c-Jun activation may lead to abnormal cell proliferation and precancerous lesion formation [39].

To further validate the findings in network pharmacology, we established an in cellular model using MNNG, which were prominently characterized by EMT. MNNG, a known potent carcinogen, induces intracellular DNA damage and mutation and inhibits the DNA repair system, thus contributing to a stable CAG model [40]. Our study revealed that PC was able to inhibit the EMT process in MC cells. First, we observed that the treatment of MC cells with PC effectively inhibited their proliferation, migration, invasion, and the EMT process. Subsequently, we performed the detection of c-Fos and c-Jun protein expression. Mechanistically, PC was shown to attenuate EMT progression via c-Fos/c-Jun pathway modulation in vitro. This anti-EMT efficacy was corroborated in MNNG-induced CAG rats with PC treatment significantly ameliorating gastric pathology. The results of Western blot experiments performed on gastric tissues were consistent with those obtained from cellular experiments.

The main feature of CAG is inflammatory infiltration [3,41]. Chronic inflammatory milieu critically drives gastritis-to-GC progression with a strategic attenuation of inflammatory cascades potently abrogating CAG pathogenesis [42]. Studies have shown that IL-6, TNF-α and IL-1β play important roles in the pathogenesis of CAG [43]. Pro-inflammatory factors such as IL-6, TNF-α and IL-1β are able to directly damage the structure and function of epithelial cells, which puts the gastric mucosal epithelial cells in a hyperproliferative state and leads to an increase in the expression of Ki67 [44]. However, excessive proliferation may trigger abnormal cell division, which may increase the risk of precancerous lesions [45]. The structural and functional impairment of epithelial cells leads to an increased permeability of the epithelial barrier, which in turn triggers cellular stress and initiates the expression of the tumor suppressor gene P53 [46]. In addition, inflammatory factor activates immune cells, which releases more inflammatory mediators in response to inflammation [47], further aggravating gastric mucosal damage. In the context of CAG, the pro-inflammatory environment might inhibit SOX2 expression [48], leading to impaired stem cell function as well as decreased gastric mucosal repair. This process may further exacerbate gastric mucosal damage and make it more susceptible to carcinogenesis.

PG I and PG II, as pepsin precursors, serve as sensitive biomarkers of gastric mucosal integrity [49]. In CAG, the levels of PG I and PG II are markedly reduced. This is primarily attributed to the damage and degeneration of gastric mucosal glands, which lead to a decline in the secretory function. In addition, G17 is a gastrointestinal hormone that promotes gastric acid secretion [50]. However, when the gastric body is in a state of insufficient gastric acid secretion due to atrophy, the level of G17 might increase through a negative feedback mechanism [51]. Studies have shown that PC significantly reduced the level of G17 while increasing the expression of PG I and PG II, thereby regulating the physiological function of the stomach. This regulatory effect helps to restore the normal function of the gastric mucosa and improve the problem of insufficient gastric acid secretion.

## 4. Materials and Methods

### 4.1. Preparation of PC Extract

PC was obtained from the Pharmacy at Hebei Provincial Hospital of Traditional Chinese Medicine, production no: 203601. The herbs were soaked in distilled water for 30 min and then reflux extracted 3 times (2 h each). We concentrated the extract and used it in the next experiment.

### 4.2. Chemical Composition of PC Extract-Containing Serum Based on UPLC-Q-Extractive Orbitrap MS/MS

The analytical instrument used in this experiment is an LC-MS system composed of an ACQUITY UPLC I-Class plus (Waters, Shanghai, China) ultra-high-performance liquid chromatography system coupled with a QE high-resolution mass spectrometer (Thermo Fisher Scientific, Waltham, MA, USA). Chromatographic separation was achieved on an HSS T3 column (100 × 2.1 mm, 1.8 μm) using a binary mobile phase of 0.1% formic acid aqueous solution (A) and acetonitrile (B). The column temperature was maintained at 45 °C with a flow rate of 0.35 mL/min, and the injection volume was 5 μL. UV absorption was monitored via a photodiode array (PDA) across 210–400 nm.

The mass spectrometry scan parameters were set as follows: ionization mode, heated electrospray (HESI) with dual polarity switching; acquisition mode, data-dependent acquisition (DDA) including full MS scans (*m*/*z* 100–1500) followed by MS^2^ scans of the top eight most intense ions. Full MS resolution was set to 70,000, and MS/MS resolution was set to 17,500. The ion source parameters were optimized as follows: spray voltage, 3800 V; capillary temperature, 320 °C; Aux gas heater temperature, 350 °C; NCE/stepped NCE was set at 10, 20, and 40 eV.

The quality control samples (QC) were prepared by mixing and centrifuging the supernatant of the drug administration group and the prototype drug in equal volumes (the concentration of the prototype drug in the QC samples was consistent with the concentration of the prototype drug in the sample solution). The detailed parameters are in Supplementary Table S6.

### 4.3. Cell Culture

Human gastric epithelial cells (GES-1) were procured from iCell Bioscience (Shanghai, China) and cultured in complete RPMI 1640 medium supplemented with 10% fetal bovine serum (FBS).

GES-1 cells were treated with 2 × 10^−5^ mol/l MNNG in complete medium for 24 h. MNNG-containing medium was replaced with fresh RPMI 1640 medium supplemented with 10% FBS to terminate chemical exposure. Significant cell death was observed within one week. Surviving cells were subsequently passaged upon reaching 80–90% confluence to establish the MC cell model. Third-passage MC cells were then utilized for subsequent experiments.

### 4.4. Cell Viability Assay

MC cell viability under PC extract treatment was quantified via CCK-8 assay. Cells (5 × 10^3^/well) were plated in 96-well plates and exposed to gradient concentrations of PC extract-supplemented serum. Following 24/48/72 h incubation, 10 μL of CCK-8 reagent was introduced to each well. Optical density measurements at 450 nm were recorded using a microplate reader to determine proliferative responses.

### 4.5. Flow Cytometry Analysis of Apoptosis and Cell Cycle

Cell apoptosis was analyzed using an Annexin V-FITC/PI apoptosis detection kit (liankebio, Hangzhou, China), and cell cycle distribution was assessed with a cell cycle analysis kit, both according to the manufacturer’s instructions. The stained cells were analyzed by a flow cytometer.

### 4.6. Cell Wound Scrape Assay

MC cells (2 × 10^5^/well) achieved confluency in 6-well plates using RPMI 1640 medium supplemented with 10% FBS. Post-scratching with a 200 μL tip, PC extract-containing medium was applied. Wound-healing progression was microscopically documented at 0/24/48 h.

### 4.7. Morphological Analysis of Apoptosis

MC cells were stained with Hoechst 33342 (Solarbio, Beijing, China): 1 mL of solution was added per well and incubated at 37 °C for 20 min; then, the staining solution was discarded, and the wells were washed twice with PBS to facilitate fluorescence detection.

### 4.8. Transwell^®^ Migration and Invasion Assay

In vitro cell migration and invasion were evaluated via Transwell® assays (Corning Incorporated, Corning, NY, USA) with 8 μm pore membranes. Invasion-specific assessments utilized Matrigel-coated inserts. Following serum starvation, 1 × 10^5^ cells were plated in the upper compartment, while the lower chamber contained 20% FBS chemoattractant. After 24 h incubation, membranes were methanol-fixed and stained with 0.1% crystal violet, and traversed cells were enumerated via light microscopy.

### 4.9. Colony Formation Assay

MC cells were plated in 6-well culture dishes (1 × 10^3^ cells/well) and allowed to adhere. After 24 h, adherent cells were exposed to serum containing PC extract for 24 h. The PC-supplemented serum was then refreshed with complete medium every 48 h over a 10-day treatment period. Colonies were finally fixed and stained with 0.5% crystal violet for quantitative morphometric analysis.

### 4.10. Animals

Sixty male Wistar rats (7 weeks old, 180–220 g) were sourced from SiPeiFu Biotechnology Co., Ltd. (Beijing, China) The ethics Committee of Hebei College of Traditional Chinese Medicine approved the study (DWLL202304006). The rats were divided into six groups based on weight: control (Ctrl), CAG, Vitacoenzyme tablets (Vitac, 0.315 g/kg), and three PC dosage groups (PC-L: 0.63 g/kg, PC-M: 1.26 g/kg, PC-H:2.52 g/kg) with 10 rats per group.

### 4.11. Enzyme-Linked Immunosorbent Assay

Serum concentrations of IL-1β, IL-6, TNF-α, PG I, PG II, and G 17 in rats were quantified using species-specific Enzyme-linked Immunosorbent Assay kits (ZCiBio, Shanghai, China), with catalog numbers as follows: IL-1β (ZC-36391), IL-6 (ZC-36404), TNF-α (ZC-37624), PG I (ZC-37308), PG II (ZC-37309), and G17 (ZC-55591).

### 4.12. HE, AB-PAS, and IHC Staining

Gastric tissues were fixed in formalin, dehydrated, embedded in paraffin, sliced to 4 μm, stained with hematoxylin and eosin staining (HE) and AB-PAS, and observed under a microscope. Paraffin sections of gastric tissues were stained with anti-Ki67 (28074-1-AP), anti-P53 (10442-1-AP), anti-SOX2 (66411-1-Ig) for immunohistochemistry (IHC) and then observed under a microscope (Wuhan Sanying Biotech Co., Ltd., Wuhan, China).

### 4.13. Western Blot Analysis

Protein lysates were isolated with RIPA buffer (Solarbio, Beijing, China) and quantified via BCA assay. SDS-PAGE electrophoretic separation was followed by semi-dry transfer onto PVDF membranes (Merck Millipore, Darmstadt, Germany). Membranes were sequentially incubated with species-matched primary/secondary antibodies with signal acquisition performed on a ChemiDoc™ MP Imaging System (Bio-Rad Laboratories, Hercules, CA, USA). The following antibodies were employed: c-Fos (66590-1-Ig), c-Jun (24909-1-AP), N-cadherin (22018-1-AP), E-cadherin (20874-1-AP), and Vimentin (10366-1-AP) (Sanying Biotechnology, Wuhan, China).

### 4.14. Network Pharmacology Analysis and Molecular Docking

The primary components of PC extract-containing serum were analyzed using UHPLC-Q-Orbitrap-MS/MS, and their CAS numbers were entered into PubChem (https://pubchem.ncbi.nlm.nih.gov/, accessed on 28 November 2023). These were imported into PharmMapper (https://lilab-ecust.cn/pharmmapper/submitfile.html, accessed on 29 November 2023), SuperPred (https://prediction.charite.de/, accessed on 29 November 2023), Swiss TargetPrediction (http://swisstargetprediction.ch/, accessed on 30 November 2023), and TCMSP (https://old.tcmsp-e.com/tcmsp.php, accessed on 30 November 2023) databases to predict and filter PC-related targets. CAG-related targets were identified using PharmGkb (https://www.pharmgkb.org/, accessed on 1 December 2023), OMIM (https://www.omim.org/, accessed on 1 December 2023), DisGeNET (https://disgenet.cn/, accessed on 1 December 2023), and GeneCards (https://www.genecards.org/, accessed on 1 December 2023) databases with “chronic atrophic gastritis” as a keyword. PC-active ingredient targets were cross-referenced with CAG-related targets using a Venn diagram. A protein–protein interaction (PPI) network was constructed by integrating the intersecting targets into the STRING database (https://cn.string-db.org/, accessed on 2 December 2023). The following steps were carried out: click “search”, select “Multiple proteins”, and input the gene name list into the name box. In the “organisms” box, select the Homo species and then click “continue”. A PPI network of PC–CAG overlapping targets was established with a confidence threshold set at 0.9. Subsequently, isolated genes without interactions were removed, and the network was visualized using Cytoscape v3.9.1.

The PPI network was constructed and analyzed in Cytoscape. A dual-phase topological screening was then performed using the CytoNCA plugin. Core genes were identified through sequential filtering based on six centrality metrics: betweenness centrality (BC), degree centrality (DC), closeness centrality (CC), eigenvector centrality (EC), local average connectivity (LAC), and network centrality (NC). In both screening phases, nodes with values exceeding the median across all parameters were retained.

The ClusterProfiler package in R was used to analyze GO functions and enrich KEGG pathways. Statistical analysis was performed using R software (version 4.0.3) with results deemed statistically significant when the *p*-value was less than 0.05. Furthermore, to visualize the interaction results of components and the outcomes of pathways, we utilized Cytoscape to create a network. The top five components of this network ranked by degree may be the active components of PC in the treatment of CAG

The GSE2669 dataset was retrieved from the Gene Expression Omnibus (GEO, https://www.ncbi.nlm.nih.gov/geo/, accessed on 3 December 2023) repository. Utilizing the Limma computational pipeline in R, we identified differentially expressed mRNAs under stringent criteria (adjusted *p* < 0.05, |log2FC| > 1). Subsequent functional enrichment analyses (GO/KEGG) were executed following the identical analytical framework outlined previously. Core therapeutic targets of PC against CAG were determined through the intersectional analysis of bioinformatics predictions and network pharmacology screening results.

Molecular docking between PC active compounds and key targets (c-Fos/c-Jun) was implemented using AutoDockTools 1.5.6 and AutoDock Vina 4.2. Ligand structures retrieved from the PubChem database underwent 3D optimization via ChemOffice14.0 energy minimization, which was followed by hydrogenation and PDBQT format conversion in AutoDockTools. Meanwhile, target crystal structures from RCSB PDB were processed in PyMOL 2.5.2 to remove solvent, add hydrogens, and define binding pockets. Active site-specific docking grids generated in AutoDockTools and exported as PDBQT files were subsequently employed for semi-flexible docking in AutoDock Vina 1.2.3 with binding affinities quantified by free energy calculations. Final binding modes and molecular interactions were visualized and analyzed using PyMOL and LigPlot^+^ 2.2.9.

### 4.15. Statistical Analysis

Dataset variability was quantified through mean ± SEM measurements, while hypothesis testing procedures were executed in GraphPad Prism 10.1.2. Between-group analyses involving two datasets adopted parametric *t*-tests with one-way ANOVA serving as the inferential framework for experiments containing three or more treatment arms. All probabilistic inferences respected the conventional 5% significance level (two-tailed).

## 5. Conclusions

PC attenuated EMT progression via the targeted modulation of the c-Fos/c-Jun pathway axis, thereby ameliorating CAG pathology. Our findings comprehensively delineated the pharmacodynamic basis of PC against CAG, establishing a mechanistic framework to guide its clinical translation.

## Figures and Tables

**Table 1:** Identification results of chemical components in PC extract-containing serum by UHPLC-Q- Orbitrap-MS/MS.

No.	Name	Formula	Annot. DeltaMass [ppm]	*m*/*z*	RT [min]	Adducts	Ion Mode
MOL01	(+)-Afzelechin	C_15_H_14_O_5_	0.78	319.0825	6.29	M+FA-H	NEG
MOL02	(Z)-Akuammidine	C_21_H_24_N_2_O_4_	−3.48	386.2062	3.96	M+NH_4_	POS
MOL03	2,6-Dimethoxyphenol	C_14_H_18_O_10_	0.95	327.0725	2.59	M-H_2_O-H	NEG
MOL04	3′,4′-Dihydroxyacetophenone	C_14_H_16_O_9_	0.77	327.0724	3.77	M-H	NEG
MOL05	4,5-Dimethoxycanthin-6-one	C_14_H_8_N_2_O_6_S	−0.15	331.003	5.04	M-H	NEG
MOL06	7-Hydroxyisoflavone	C_16_H_12_O_7_S	0.43	697.0683	5.51	2M+H	POS
MOL07	15-Hydroxydehydroabietic acid	C_20_H_28_O_4_	0.04	350.2326	6.48	M+NH_4_	POS
MOL08	Alfacalcidol	C_27_H_44_O_3_	−0.37	417.3362	11.29	M+H	POS
MOL09	Angelol A	C_26_H_32_O_13_	0.08	570.2182	5.76	M+NH_4_	POS
MOL10	Chicoric acid	C_9_H_10_O_7_S	0.47	261.0076	3.76	M-H	NEG
MOL11	Columbianadin	C_19_H_20_O_6_	0.71	343.119	8.07	M-H	NEG
MOL12	Guaiacin	C_26_H_32_O_11_	−3.47	559.1558	5.23	M+K	POS
MOL13	Icaritin	C_27_H_28_O_12_	−4.46	527.1524	8.83	M+H-H_2_O	POS
MOL14	Kaurenoic acid	C_20_H_30_O_3_	0.43	301.2163	9.99	M+H-H_2_O	POS
MOL15	Methyl indole-3-carboxylate	C_16_H_17_NO_9_	0.38	368.0977	4.35	M+H	POS
MOL16	m-Methoxyphenol	C_7_H_8_O_5_S	1.04	203.0022	4.52	M-H	NEG
MOL17	Nuciferine	C_19_H_21_NO_3_	2.12	623.3129	5.62	2M+H	POS
MOL18	6-hydroxy-7-(hydroxymethyl)-3-(1H-pyrrol-3-yl) isochromen-1-one	C_14_H_11_NO_4_	1.32	302.0674	4.66	M+FA-H	NEG
MOL19	Proxyphylline	C_10_H_12_N_4_O_3_	−4.95	217.0719	4.18	M-H_2_O-H	NEG
MOL20	Trametenolic acid	C_30_H_48_O_4_	−4.02	495.3426	11.92	M+Na	POS
MOL21	Gamma-l-glutamyl-l-tyrosine	C_14_H_18_N_2_O_6_	−1.03	311.1234	2.47	M+H	POS
MOL22	2-Heptadecanone	C_17_H_34_O	0.08	299.2592	13.19	M+FA-H	NEG

Note: NEG/POS: negative/positive ionization mode in mass spectrometry; MOL: molecule.

## Data Availability

The data presented in this study are available on request from the corresponding author. The data are not publicly available due to privacy.

## Supplementary Materials

The following supporting information can be downloaded at: https://www.mdpi.com/article/10.3390/ph18040537/s1, Figure S1 Network pharmacological analysis. Table S1 The difference analysis results of normal and gastric cancer. Table S2 The difference analysis results of normal and gastritis. Supplementary Table S3 The network of the top 5 components in degree value about Figure S1G. Table S4 The binding energy of the top 5 components. Table S5 Composition of PC. Table S6 Technical data and the manufacturer ACQUlTY UPLC. Table S7 Gastric cancer vs normal Deg Genes. Table S8 Chronic gastritis vs normal Deg Genes. Table S9 List of English acronyms. Table S10 CytoNCA score.
