# Supplementary material for: Investigation of c-Fos/c-Jun Signaling Pathways in Periostracum Cicadae’s Inhibition of EMT in Gastric Tissue
Source: Pharmaceuticals (Basel). 2025 Apr 7;18(4):537. doi: 10.3390/ph18040537 (PMC12030197; doi:10.3390/ph18040537)
Supplement: Supplementary file 1 [file pharmaceuticals-18-00537-s001.zip › Supplementary Table S3 The network of the top 5 components in degree value about Figure S1G.pdf]

Table S3. The network of the top 5 components in degree value about Figure S1G

type	shared name	Degree	BetweennessCentrality	ClosenessCentrality	Eccentricity	NeighborhoodConnectivity
**MOL**	**MOL12**	**82**	**0.08180771727067439**	**0.4213564213564214**	**3**	**10.788732394366198**
**MOL**	**MOL21**	**77**	**0.14253904842719628**	**0.4213564213564214**	**3**	**7.633802816901408**
**MOL**	**MOL02**	**74**	**0.042013626786813967**	**0.40387275242047027**	**3**	**11.035714285714286**
**MOL**	**MOL10**	**70**	**0.06544711017238306**	**0.4072524407252441**	**3**	**10.423728813559322**
**MOL**	**MOL11**	**65**	**0.05951566786424217**	**0.4072524407252441**	**3**	**10.76271186440678**
MOL	MOL14	63	0.08561449054812388	0.40499306518723993	3	9.508771929824562
MOL	MOL17	62	0.11282250569050395	0.40387275242047027	3	7.714285714285714
MOL	MOL15	59	0.05706743206109076	0.4005486968449931	3	10.037735849056604
pathway	hsa05417	56	0.03967309768898481	0.4027586206896552	3	12.472727272727273
MOL	MOL08	54	0.03564812029183993	0.38985313751668893	3	12
MOL	MOL13	54	0.032200626855447285	0.39406207827260464	3	11.170212765957446
MOL	MOL05	52	0.04326470600876725	0.39727891156462586	3	10.44
MOL	MOL06	50	0.022238399487178092	0.3857331571994716	3	11.487179487179487
MOL	MOL20	50	0.044162181853736	0.39300134589502017	3	10.130434782608695
pathway	hsa05208	45	0.03205262475190829	0.3908969210174029	3	12.477272727272727
medicinal material	PC	44	0.024732959856281416	0.5	4	45.590909090909
gene	NFKB1	42	0.051879862562519995	0.5289855072463768	4	40.39473684210526
MOL	MOL07	42	0.031416391847814154	0.38778220451527223	3	11.609756097560975
MOL	MOL19	41	0.038745084468065974	0.3826998689384011	3	11.416666666666666

pathway	hsa05171	41	0.018760548834070363	0.38675496688741723	3	13.975
MOL	MOL22	40	0.04157229182226617	0.3826998689384011	3	10.444444444444445
Disease	CAG	40	0.0027890281398515343	0.3459715639810427	4	33.4
pathway	hsa05205	40	0.01675988944451908	0.3857331571994716	3	13.051282051282051
MOL	MOL09	39	0.02468912988997236	0.38370565045992117	3	12.27027027027027
pathway	hsa05161	39	0.012820406539602406	0.3847167325428195	3	15.736842105263158
MOL	MOL04	38	0.03723525580480443	0.3787289234760052	3	11.28125
pathway	hsa05163	38	0.01253478297435584	0.38370565045992117	3	14.945945945945946
pathway	hsa05418	37	0.01690442484267902	0.3826998689384011	3	13.5
pathway	hsa05167	37	0.012299521436027498	0.3826998689384011	3	15.555555555555555
pathway	hsa04933	34	0.012011549152270606	0.37971391417425226	3	14.727272727272727
pathway	hsa05164	34	0.010488185027373526	0.37971391417425226	3	14.363636363636363
MOL	MOL16	33	0.02615403846788152	0.3748395378690629	3	12.107142857142858
MOL	MOL18	33	0.01827555946932901	0.3767741935483871	4	10.03225806451613
pathway	hsa04380	33	0.008545960186866342	0.3787289234760052	3	15.59375
MOL	MOL01	32	0.010256061947183742	0.3758043758043758	3	13.137931034482758
pathway	hsa05142	31	0.007826200666614349	0.3767741935483871	3	16
pathway	hsa04668	31	0.008489756702079233	0.3767741935483871	3	15.766666666666667
gene	MAPK1	30	0.020360154683800997	0.45768025078369906	4	39
pathway	hsa05145	30	0.010452123487131827	0.3758043758043758	3	14.068965517241379
gene	MAPK14	28	0.01839901894145839	0.4478527607361963	4	40.214285714285715
MOL	MOL03	28	0.0071925661193598086	0.3729246487867177	3	14.615384615384615

pathway	hsa01521	28	0.006712908938904939	0.3738796414852753	3	13.962962962962964
pathway	hsa05215	28	0.009859981239808193	0.3738796414852753	3	14.666666666666666
pathway	hsa04620	28	0.004800418340565636	0.3738796414852753	3	17.51851851851852
pathway	hsa05212	27	0.0063358527404895	0.3729246487867177	3	15.692307692307692
pathway	hsa05235	26	0.004323237556726381	0.37197452229299366	3	17.84
gene	TTR	25	0.007186240655622591	0.41595441595441596	4	43.2
pathway	hsa05140	25	0.006033948954518293	0.36915297092288246	5	14.208333333333334
gene	CA2	22	0.012246129784471883	0.44376899696048633	4	43.611111111111114
gene	APEX1	22	0.02216169417310289	0.4915824915824916	4	46.04761904761905
gene	CHUK	22	0.009605804244638364	0.4147727272727273	4	37
gene	MAPK8	21	0.00898431196939833	0.41126760563380277	4	38.76190476190476
gene	NFE2L2	21	0.012699287821029243	0.45201238390092874	4	46.8235294117647
gene	GSK3B	21	0.011377262856928487	0.43323442136498513	4	43.333333333333336
gene	SRC	21	0.01244028067049315	0.4451219512195122	4	45.1578947368421
gene	PIK3CB	21	0.0062338549247231406	0.38421052631578945	4	34.76190476190476
gene	PIK3CD	21	0.005574440422714375	0.38726790450928383	4	35.857142857142854
gene	AR	20	0.008205774316582436	0.4294117647058824	4	44.15384615384615
gene	PIK3CA	20	0.004336578944861067	0.37340153452685426	4	34.7
gene	MAPK3	20	0.003423461129521117	0.36500000000000005	4	35.15
gene	NTRK3	19	0.01694410846858589	0.47557003257328984	4	47
gene	IKBKG	19	0.004151103334836829	0.381201044386423	4	36.89473684210526
gene	STAT1	19	0.005269196625880541	0.38522427440633245	4	36

gene	AKT1	19	0.003076018312061612	0.3604938271604938	4	34.26315789473684
gene	SLC2A1	18	0.01399744885732058	0.45625000000000004	4	45.529411764705884
gene	TLR4	18	0.013573979535936332	0.44376899696048633	4	41.611111111111114
gene	PRKCA	17	0.004878022455811984	0.3882978723404255	4	39.4
gene	STAT3	17	0.0073888946392239	0.40782122905027934	4	39.875
gene	MAP2K1	17	0.004517572302968487	0.38219895287958117	4	36.35294117647059
gene	FOS	17	0.007153502204389619	0.39890710382513667	4	38.88235294117647
gene	NQO1	16	0.004806465551378048	0.3882978723404255	4	40.5
gene	HIF1A	16	0.006696204654249912	0.41359773371104813	4	44.69230769230769
gene	GSTP1	16	0.00943317047339693	0.4424242424242424	4	50
gene	CYP3A4	16	0.011686281898243892	0.45482866043613707	4	49.06666666666667
gene	JUN	16	0.003877634239397453	0.38421052631578945	4	39.5625
gene	TGFBR1	15	0.005377953812487496	0.4089635854341737	4	45.714285714285715
gene	TNF	15	0.00274518547881735	0.35960591133004927	4	36
gene	PTGS1	14	0.006611032987765523	0.4219653179190751	4	49.09090909090909
gene	VDR	14	0.0013619539321009242	0.3526570048309179	4	47.142857142857146
gene	PTGS2	14	0.005601152465354367	0.40443213296398894	4	43.45454545454545
gene	MAP2K2	14	0.004071226619643275	0.38522427440633245	4	37.642857142857146
gene	IL1B	14	0.006759194170508986	0.4	4	40.07142857142857
gene	PRKCB	13	0.003119439222808861	0.37150127226463103	4	36.76923076923077
gene	MIF	13	0.006442567385819759	0.4124293785310735	4	47.4
gene	MMP9	13	0.004691802469767974	0.40782122905027934	4	46.25

gene	NOX1	13	0.006137690963033789	0.41011235955 05618	4	43.083333333333336
gene	CASP3	13	0.0027959951552393772	0.37628865979381443	4	42
gene	JAK1	13	0.001314597187615438	0.3411214953271028	4	33.30769230769231
gene	EGFR	12	0.0024332453793385255	0.37628865979381443	4	40.083333333333336
gene	TNFRSF1A	12	0.001627595221197075	0.3484486873508353	4	36.416666666666664
gene	IL6	12	0.0025587884010994494	0.3501199040767386	4	36.833333333333336
gene	ALOX5	11	0.0021901711875454157	0.37340153452685426	4	41.55555555555556
gene	ADAM17	11	0.002287011244130659	0.37340153452685426	4	41.5
gene	MET	11	0.0026196494304412685	0.38421052631578945	4	44.3
gene	KDM1A	11	0.004771545581279713	0.4	4	48.9
gene	KDR	11	0.004026345682420224	0.40109890109890106	4	47.81818181818182
gene	PTPN11	11	0.004164905072020248	0.38726790450928383	4	42.72727272727273
gene	FPR2	11	0.006365280684338249	0.4207492795389049	4	50.72727272727273
gene	PPARG	11	0.00492599286326979	0.4207492795389049	4	51.3
gene	MMP2	11	0.0054161332493990605	0.4256559766763849	4	50.8
gene	TGFB2	11	0.0018835581158192134	0.35696821515892424	4	36.45454545454545
gene	TLR2	11	0.002911332829589787	0.369620253164557	4	37.63636363636363
gene	MMP3	10	0.0022456310519763122	0.37340153452685426	4	41.55555555555556
gene	PLAU	10	0.0031348704834692773	0.3792207792207792	4	41.6
gene	ITGB1	10	0.0044791139129679415	0.40782122905027934	4	48.55555555555556
gene	PDE5A	10	0.0016608772733231127	0.3659147869674185	4	49.375
gene	IL2	10	0.0033072740758186264	0.3882978723404255	4	44.2

gene	MTOR	10	0.0018009558252701527	0.3435294117647059	4	36.3
gene	CXCL8	10	0.0018553640520553385	0.3509615384615385	4	40
gene	SOD2	9	0.002635370105800932	0.3978201634877384	4	50.888888888888886
gene	VEGFA	9	7.205880013695781E-4	0.3288288288288288	4	34.111111111111114
gene	LGALS3	9	8.45239812972308E-4	0.3348623853211009	4	38.57142857142857
gene	ACE	9	0.003000303337656573	0.3862433862433862	4	48.142857142857146
gene	PGR	9	0.0013334013749388392	0.3543689320388349	4	48.714285714285715
gene	ADORA2B	9	0.0016933818221405693	0.35872235872235875	4	45.857142857142854
gene	ALB	9	0.0032614598577808324	0.3945945945945946	4	49.44444444444444
gene	SERPINE1	9	0.002219133562233874	0.36318407960199006	4	39.44444444444444
gene	MMP12	9	0.002705013855171184	0.38020833333333337	4	47.42857142857143
gene	IGF1	9	0.002723987236310733	0.38320209973753283	4	44.44444444444444
gene	SELE	9	0.0019449139052078685	0.37628865979381443	4	47.888888888888886
gene	CFTR	9	0.002523124399411968	0.38726790450928383	4	50.125
gene	BAX	9	7.234056645923132E-4	0.3310657596371882	4	36.44444444444444
gene	CASP9	9	0.0017748926977087082	0.3492822966507177	4	39.111111111111114
gene	F2	8	0.0014972609548149754	0.36318407960199006	4	45.375
gene	CTNNA1	8	0.0011656606085078361	0.3509615384615385	4	43.875
gene	CHRM3	8	0.0025991411291474142	0.3862433862433862	4	58
gene	ABL1	8	0.0013310402073787113	0.36228287841191065	4	50.57142857142857
gene	MMP8	8	0.0028926971850738712	0.38219895287958117	4	48.875
gene	HDAC2	8	0.002673378680826282	0.3668341708542714	4	42.625

gene	CTSK	8	0.001222682654043968	0.36318407960199006	4	46.57142857142857
gene	CCL5	8	0.0018265806446068741	0.35784313725490197	4	40.875
gene	TYK2	8	5.662065094442336E-4	0.3325740318906606	4	36.75
gene	HDAC9	7	0.0013877761982490754	0.35180722891566263	4	47.166666666666664
gene	SYK	7	0.0010033494517742334	0.35784313725490197	4	44.285714285714285
gene	CYP2C9	7	9.897865226821487E-4	0.37055837563451777	4	55.333333333333336
gene	PARP1	7	0.001067879097641862	0.3604938271604938	4	54.333333333333336
gene	XIAP	7	0.0017442513052755844	0.37340153452685426	4	44.857142857142854
gene	TGFBR2	7	7.41527275727524E-4	0.3318181818181818	4	35.57142857142857
gene	MMP1	7	0.0018864652595235432	0.38320209973753283	4	58.166666666666664
gene	ICAM1	7	5.979835550519204E-4	0.33874709976798145	4	40.285714285714285
gene	NOS2	7	0.0011366501282061576	0.35960591133004927	4	43.333333333333336
gene	NCF2	7	0.001578868153964896	0.3613861386138614	4	45.42857142857143
gene	CYBA	7	0.001578868153964896	0.3613861386138614	4	45.42857142857143
gene	NCF1	7	0.001578868153964896	0.3613861386138614	4	45.42857142857143
gene	BRAF	7	5.775640600866415E-4	0.3333333333333333	4	38.857142857142854
gene	FASLG	7	8.630174642328685E-4	0.33874709976798145	4	41.285714285714285
gene	XDH	6	1.8798546419832948E-4	0.3187772925764192	4	43.5
gene	ELANE	6	0.0015755833144746308	0.35523114355231145	4	42.666666666666664
gene	ABO	6	0.0010869339140605503	0.35609756097560974	4	49.833333333333336
gene	MDM2	6	6.24800485329799E-4	0.33640552995391704	4	41.333333333333336
gene	MMP7	6	0.0018452213528782717	0.3686868686868687	4	53

gene	CYP1A1	6	0.0011642777448724038	0.35180722891566263	4	41.5
gene	SNCA	6	0.002984211860106817	0.3792207792207792	4	51.166666666666664
gene	CMA1	6	0.0014848141111074385	0.35872235872235875	4	49.166666666666664
gene	BIRC7	6	0.0020529638459908872	0.38219895287958117	4	49
gene	ACACA	6	0.0012074244269589791	0.3427230046948357	4	47
gene	PTK2	6	5.9655202970469E-4	0.33874709976798145	4	45
gene	RPS6KB1	6	3.582734477703561E-4	0.32158590308370044	4	35.5
gene	CYBB	6	0.0012382415913461326	0.3543689320388349	4	46.5
gene	F13A1	5	6.141450062910372E-4	0.3340961098398169	4	39.2
gene	ACVRL1	5	0.0010878933631388984	0.35351089588377727	4	52.4
gene	S100A9	5	0.0010974516758870698	0.35523114355231145	4	53.2
gene	DAPK1	5	4.455071156264387E-4	0.33796296296296297	4	48.4
gene	HMOX1	5	0.0010538477150499274	0.3484486873508353	4	45.8
gene	ODC1	5	6.010499420625087E-4	0.34515366430260047	4	49.8
gene	TH	5	0.0017335390985389646	0.35523114355231145	4	54.5
gene	CTSB	5	0.0010599182630624555	0.3501199040767386	4	50
gene	LCN2	5	0.0016501620002084012	0.36228287841191065	4	54.4
gene	CTSL	5	0.001149853194556536	0.3604938271604938	4	48.4
gene	ADORA2A	5	6.802092913386321E-4	0.32808988764044944	4	50.333333333333336
gene	NCF4	5	9.213722074090205E-4	0.3492822966507177	4	47.6
gene	CASP1	5	0.0011365385924578934	0.35609756097560974	4	49
gene	MAP3K5	5	5.833129116957351E-4	0.32516703786191536	4	40.2

gene	CNR1	4	0.0015031406962646389	0.3492822966507177	4	48
gene	ANXA5	4	3.1415417775314296E-4	0.3230088495575221	4	45.5
gene	SERPINA1	4	5.384875358999607E-4	0.3318181818181818	4	48
gene	HSPA1A	4	4.7991730794839406E-4	0.3333333333333333	4	45.25
gene	INS	4	0.0011539294980614193	0.34679334916864607	4	47.5
gene	CXCR4	4	8.818074615914492E-4	0.3484486873508353	4	48.5
gene	KAT2B	4	4.370291525670029E-4	0.32589285714285715	4	45
gene	NLRP3	4	2.8183984595777056E-4	0.3230088495575221	4	44.5
gene	ALDH2	4	1.7427236500180222E-4	0.3237250554323725	4	56.333333333333336
gene	ACP1	4	2.465648456890573E-4	0.3146551724137931	4	39.666666666666664
gene	AURKA	4	1.8693456905507488E-4	0.31947483588621445	4	47.666666666666664
gene	ADH1B	4	5.07678325562671E-4	0.31670281995661603	4	37.75
gene	FHIT	4	4.1347542751880677E-4	0.3333333333333333	4	50.5
gene	BLVRB	4	5.607360794892625E-4	0.33563218390804594	4	49
gene	B3GAT1	4	8.924325703707643E-4	0.3543689320388349	4	59.5
gene	F3	4	9.403575444275304E-4	0.3509615384615385	4	54.75
gene	F2R	4	0.0014011565730875412	0.3613861386138614	4	58.25
gene	GPR35	4	5.066632853290598E-4	0.3244444444444445	4	44.5
gene	SELP	4	3.820200159369485E-4	0.3333333333333333	4	49.25
gene	GSTM1	4	8.991695168730649E-4	0.35609756097560974	4	55.5
gene	CYP1A2	4	4.322468592559837E-4	0.3325740318906606	4	43
gene	PDGFRB	4	2.804441419466805E-4	0.3139784946236559	4	34.25

gene	FGF2	4	3.1207872734615523E-4	0.31808278867102396	4	39.75
gene	HLA-DRB1	4	4.254750238504654E-4	0.3244444444444445	4	39.25
gene	SLC18A2	3	1.7053543778054186E-4	0.3113006396588486	4	56
gene	CBS	3	4.8659361807897283E-4	0.3201754385964912	4	46.666666666666664
gene	HSPD1	3	3.1325685390539494E-4	0.32229580573951433	4	48.666666666666664
gene	KCNMA1	3	5.749692017749424E-4	0.3208791208791209	4	45
gene	ANXA1	3	2.512228604881217E-4	0.31808278867102396	4	51
gene	MTHFD1	3	1.5800876518773832E-4	0.3139784946236559	4	44.666666666666664
gene	ITGAL	3	1.6717615928484681E-4	0.3133047210300429	4	48.666666666666664
gene	RNASE3	3	2.1473868383489034E-4	0.32229580573951433	4	53
gene	HSPA1B	3	1.706836040784713E-4	0.32158590308370044	4	47.666666666666664
gene	PRSS1	3	4.1370008330359495E-4	0.33640552995391704	4	54.333333333333336
gene	NOD1	3	5.937533259346437E-4	0.3348623853211009	4	55.333333333333336
gene	PIK3CG	3	1.1398153324761294E-4	0.3187772925764192	4	45.333333333333336
gene	COX5A	3	3.24182168921161E-4	0.32158590308370044	4	49
gene	COX2	3	3.24182168921161E-4	0.32158590308370044	4	49
gene	AHR	3	2.529085009612501E-4	0.31533477321814257	4	41.666666666666664
gene	MMP14	3	4.1117012756273385E-4	0.33031674208144796	4	51.333333333333336
gene	RAD51	3	2.4318492651178146E-4	0.3113006396588486	4	38.333333333333336
gene	CAV3	3	3.5391536877183566E-4	0.3173913043478261	4	43.666666666666664
gene	TYMP	2	4.695607171770893E-5	0.3146551724137931	4	63.5
gene	MECOM	2	1.0272063940051449E-4	0.3080168776371308	4	53.5

gene	GPI	2	7.704892002176469E-5	0.3086680761099366	4	54.5
gene	ERCC5	2	3.6303956435370296E-5	0.2868369351669941	4	33.5
gene	BCR	2	8.301996351178694E-5	0.30103092783505153	4	49
gene	REG1A	2	2.7394798127649798E-5	0.2937625754527163	4	42
gene	MMP10	2	2.4545952245517955E-4	0.32158590308370044	4	65
gene	CAT	2	8.241162259158347E-5	0.30997876857749473	4	51.5
gene	HCRTR2	2	2.498913816141997E-4	0.316017316017316	4	57.5
gene	TPH1	2	2.0636439442729473E-4	0.32229580573951433	4	65
gene	NDUFA13	2	4.995681563646711E-5	0.3113006396588486	4	57.5
gene	NDUFS5	2	4.995681563646711E-5	0.3113006396588486	4	57.5
gene	SCN7A	2	1.478572657010694E-4	0.30103092783505153	4	46.5
gene	GATA3	2	4.7495401453341417E-4	0.32589285714285715	4	64
gene	PLA2G1B	2	7.528118134000822E-5	0.2997946611909651	4	51.5
gene	MPO	2	1.7890322668177047E-4	0.31263383297644537	4	55
gene	CYP2C19	2	7.084983955583434E-5	0.2937625754527163	4	40.5
gene	CCKBR	2	2.1233277828988984E-4	0.316017316017316	4	62
gene	CD34	2	5.105220399998071E-4	0.32158590308370044	4	63.5
gene	EGR1	2	1.4383744921140482E-4	0.3041666666666667	4	44.5
gene	OXT	2	5.105220399998071E-4	0.32158590308370044	4	63.5
gene	CRH	2	5.105220399998071E-4	0.32158590308370044	4	63.5
gene	BMPR1A	2	1.2285207971558524E-4	0.30353430353430355	4	46
gene	CASP6	2	1.0155038103296014E-4	0.3041666666666667	4	45.5

gene	ACE2	2	1.468633954731955E-4	0.316017316017316	4	55.5
gene	PAM	2	2.8171560756682653E-4	0.3113006396588486	4	53.5
gene	KLK3	2	1.516061506537912E-4	0.30997876857749473	4	49
gene	PCNA	2	1.4755884123740108E-4	0.316017316017316	4	54.5
gene	ERCC1	1	0	0.2749529190207156	4	32
gene	IGFBP1	1	0	0.2749529190207156	4	32
gene	IGFBP2	1	0	0.2749529190207156	4	32
gene	ERCC4	1	0	0.2749529190207156	4	32
gene	G6PD	1	0	0.28460038986354774	4	50
gene	BCL9	1	0	0.2796934865900383	4	41
gene	GFI1	1	0	0.28076923076923077	4	43
gene	BCL6	1	0	0.28076923076923077	4	43
gene	OAT	1	0	0.27756653992395436	4	37
gene	LTF	1	0	0.28968253968253965	4	59
gene	PLK1	1	0	0.28968253968253965	4	59
gene	PTPN22	1	0	0.28968253968253965	4	59
gene	CCNB1	1	0	0.2967479674796748	4	71
gene	IL13	1	0	0.2885375494071146	4	57
gene	S100A8	1	0	0.2885375494071146	4	57
gene	FGF4	1	0	0.2885375494071146	4	57
gene	ADIPOQ	1	0	0.2885375494071146	4	57
gene	NOTCH1	1	0	0.2885375494071146	4	57

gene	PRTN3	1	0	0.28627450980392155	4	53
gene	PTPRC	1	0	0.28627450980392155	4	53
gene	CASR	1	0	0.27289719626168224	4	28
gene	LGALS1	1	0	0.27289719626168224	4	28
gene	HRH2	1	0	0.2879684418145957	4	56
gene	SCT	1	0	0.2879684418145957	4	56
gene	OXTR	1	0	0.2879684418145957	4	56
gene	HCRT	1	0	0.2879684418145957	4	56
gene	CRHR2	1	0	0.2879684418145957	4	56
gene	NOXA1	1	0	0.2879684418145957	4	56
gene	NOXO1	1	0	0.2879684418145957	4	56
gene	GHRL	1	0	0.2879684418145957	4	56
gene	CALCA	1	0	0.2879684418145957	4	56
gene	HCRTR1	1	0	0.2879684418145957	4	56
gene	CD209	1	0	0.27392120075046905	5	31
gene	ATM	1	0	0.27703984819734345	4	36
gene	COL5A1	1	0	0.27703984819734345	4	36
gene	CHIA	1	0	0.27703984819734345	4	36
gene	CYP17A1	1	0	0.28239845261121854	4	46
gene	PRKCH	1	0	0.28239845261121854	4	46
gene	DDC	1	0	0.2967479674796748	4	71
gene	DNMT3A	1	0	0.2967479674796748	4	71

gene	CYP11A1	1	0	0.2967479674796748	4	71
gene	BDNF	1	0	0.2967479674796748	4	71
gene	ARF1	1	0	0.2967479674796748	4	71
gene	CBX7	1	0	0.2967479674796748	4	71
gene	CCKAR	1	0	0.2967479674796748	4	71
gene	IL2RA	1	0	0.2967479674796748	4	71
gene	IL23R	1	0	0.2967479674796748	4	71
gene	TGM2	1	0	0.27703984819734345	4	36
gene	ACER2	1	0	0.27703984819734345	4	36
gene	ADH7	1	0	0.27703984819734345	4	36
