# Supplementary material for: Investigation of c-Fos/c-Jun Signaling Pathways in Periostracum Cicadae’s Inhibition of EMT in Gastric Tissue
Source: Pharmaceuticals (Basel). 2025 Apr 7;18(4):537. doi: 10.3390/ph18040537 (PMC12030197; doi:10.3390/ph18040537)
Supplement: Supplementary file 1 [file pharmaceuticals-18-00537-s001.zip › Supplementary Table S4 The binding energy of the top 5 components.pdf]

Table S4. The binding energy of the top 5 components with c-FOS/c-JUN

No.	Components	Binding energy	protein
**1**	**(Z)-Akuammidine**	**-7.8**	**c-FOS**
**2**	**Chicoric acid**	**-8.4**	**c-FOS**
**3**	**Columbianadin**	**-8.3**	**c-FOS**
4	gamma-l-glutamyl-l-tyrosine	-7.3	c-FOS
5	Guaiacin	-6.9	c-FOS
**6**	**(Z)-Akuammidine**	**-7.3**	**c-JUN**
**7**	**Chicoric acid**	**-8.3**	**c-JUN**
**8**	**Columbianadin**	**-7.6**	**c-JUN**
9	gamma-l-glutamyl-l-tyrosine	-6.3	c-JUN
10	Guaiacin	-7.0	c-JUN
