# Supplementary material for: Investigation of c-Fos/c-Jun Signaling Pathways in Periostracum Cicadae’s Inhibition of EMT in Gastric Tissue
Source: Pharmaceuticals (Basel). 2025 Apr 7;18(4):537. doi: 10.3390/ph18040537 (PMC12030197; doi:10.3390/ph18040537)
Supplement: Supplementary file 1 [file pharmaceuticals-18-00537-s001.zip › Supplementary Table S6 Technical data and the manufacturer ACQUITY UPLC .pdf]

Instrument Information sheet

Instrument	Model & Specification	Manufacturer
Ultrasonic Cleaner	F-060SD	Shenzhen Fuyang Technology Group Co., Ltd.
Vortex Shaker	TYXH-I	Shanghai Hanno Instrument Co., Ltd.
Bench-top High-speed Refrigerated Centrifuge	TGL-16MS	Shanghai Luxiangyi Centrifuge Instrument Co., Ltd.
High Performance Liquid Chromatograph	ACQUITY UPLC I-Class plus	Waters
Chromatography Column	ACQUITY UPLC HSS T3 (100 mm×2.1 mm, 1.8 μm)	Waters
PDA Detector	ACQUity UPLC	Waters
High-Resolution Liquid Chromatography-Mass Spectrometer	Thermo-Obritrap-QE	

Reagent Information Sheet

**Name of reagent**	**Item No.**	**English name/alias of manufacturer**
methanol	A452-4	fisher
acetonitrile	A998-4	fisher
Formic acid	A117-50	fisher
Water	——	——

Methods

Sample Pretreatment

Experimental procedures are as follows:

Serum Sample Pretreatment:

1. Retrieve samples stored at -80°C and thaw slowly on ice. Transfer 150 μL of sample to a 1.5 mL EP tube;

2. Add 450 μL protein precipitant (methanol-acetonitrile, V:V=2:1, containing 2 μg/mL L-2-chlorophenylalanine). Vortex mix for 1 min;

3. Perform ultrasonic extraction in ice-water bath for 10 min, then let stand at -40°C for 30 min;

4. Centrifuge at 12,000 rpm (4°C) for 10 min. Let stand at -40°C for 2 h. Transfer 500 μL supernatant to LC-MS vial for drying;

5. Reconstitute with 150 μL methanol-acetonitrile-water (V:V:V=2:1:1). Vortex mix for 1 min followed by 3 min sonication;

6. Let stand at -40°C overnight;

7. Centrifuge at 12,000 rpm (4°C) for 10 min. Transfer 100 μL supernatant to LC-MS vial with footed insert for analysis.

Traditional Chinese Medicine (TCM) Pretreatment:

1. Transfer 100 μL sample to 1.5 mL EP tube;

2. Add 900 μL purified water (containing 4 μg/mL L-2-chlorophenylalanine) and dissolve at approximately 80°C;

3. Vortex mix for 2 min followed by 30 min ultrasonic extraction;

4. Centrifuge at 12,000 rpm (4°C) for 30 min;

5. Filter supernatant through 0.22 μm aqueous phase membrane. Transfer 200 μL filtrate to vial with wide insert for analysis.

Quality Control (QC) Sample Preparation:

QC samples were prepared by mixing equal volumes of supernatant from administration group and prototype group, followed by centrifugation. (The prototype group concentration in QC samples was consistent with the prototype drug concentration for instrument analysis).

LC-MS Conditions

The analytical system consisted of an ACQUITY UPLC I-Class plus ultra-performance liquid chromatograph coupled with a QE high-resolution mass spectrometer.

Chromatographic Conditions:

- Column: ACQUITY UPLC HSS T3 (100 mm×2.1 mm, 1.8 μm)

- Column temperature: 45°C

- Mobile phase:

  A: Water (containing 0.1% formic acid)

  B: Acetonitrile

- Flow rate: 0.35 mL/min

- Injection volume: 5 μL

PDA scanning range: 210-400 nm

Elution gradient information table

Time	A%	B%
0	95	5
2	95	5
4	70	30
8	50	50
10	20	80
14	0	100
15	0	100
15.1	95	5
16	95	5

Mass Spectrometry Conditions:

Ion source: HESI

Mass spectrometry signal acquisition: Performed in both positive and negative ion scanning modes

Data acquisition mode: DDA (Data-Dependent Acquisition)

Scan mode: Full MS/dd-MS^2 (TOP 8)

Table of mass spectrometry parameter information

parameters	Positive ion	Negative ion
Aux gas flow rate (Arb)	8	8
Full ms resolution	70000	70000
S-lens RF level	50	50
Aux gas heater temperature (°C)	350	350
Sheath Gas Flow Rate (Arb)	35	35
Capillary Temperature (°C)	320	320
MS/MS resolution	17500	17500
Mass range (m/z)	100-1200	100-1200
NCE/stepped NCE	10，20，40	10，20，40
Spray Voltage (V)	3800	-3000
