# Supplementary material for: Investigation of c-Fos/c-Jun Signaling Pathways in Periostracum Cicadae’s Inhibition of EMT in Gastric Tissue
Source: Pharmaceuticals (Basel). 2025 Apr 7;18(4):537. doi: 10.3390/ph18040537 (PMC12030197; doi:10.3390/ph18040537)
Supplement: Supplementary file 1 [file pharmaceuticals-18-00537-s001.zip › Supplementary Table S9 List of English acronyms.pdf]

Supplementary Table S9. Abbreviations

PC	Periostracum Cicadae
MC cells	model cells for chronic atrophic gastritis
TNF-α	Tumor Necrosis Factor-alpha
IL-6	Interleukin-6
IL-1β	Interleukin-1 beta
CAG	Chronic atrophic gastritis
G17	Gastrin 17
GO	Gene Ontology
KEGG	Kyoto Encyclopedia of Genes and Genomes
MNNG	N-methyl-N′-nitro-N-nitrosoguanidine
PG I	Pepsinogen I
PG II	Pepsinogen II
HE	Hematoxylin and Eosin staining
IHC	Immunohistochemistry
GEO	Gene Expression Omnibus
AB-PAS	Alcian Blue-Periodic Acid-Schiff staining
POS	Positive Ion Mode
NEG	Negative Ion Mode
DEGs	Differentially Expressed Genes
MOL	Molecule
Ki67	Antigen KI-67
P53	Tumor Protein p53
SOX2	SRY-box Transcription Factor
