# Supplementary figures and images for: Investigation of c-Fos/c-Jun Signaling Pathways in Periostracum Cicadae’s Inhibition of EMT in Gastric Tissue
Source: Pharmaceuticals (Basel). 2025 Apr 7;18(4):537. doi: 10.3390/ph18040537 (PMC12030197; doi:10.3390/ph18040537)

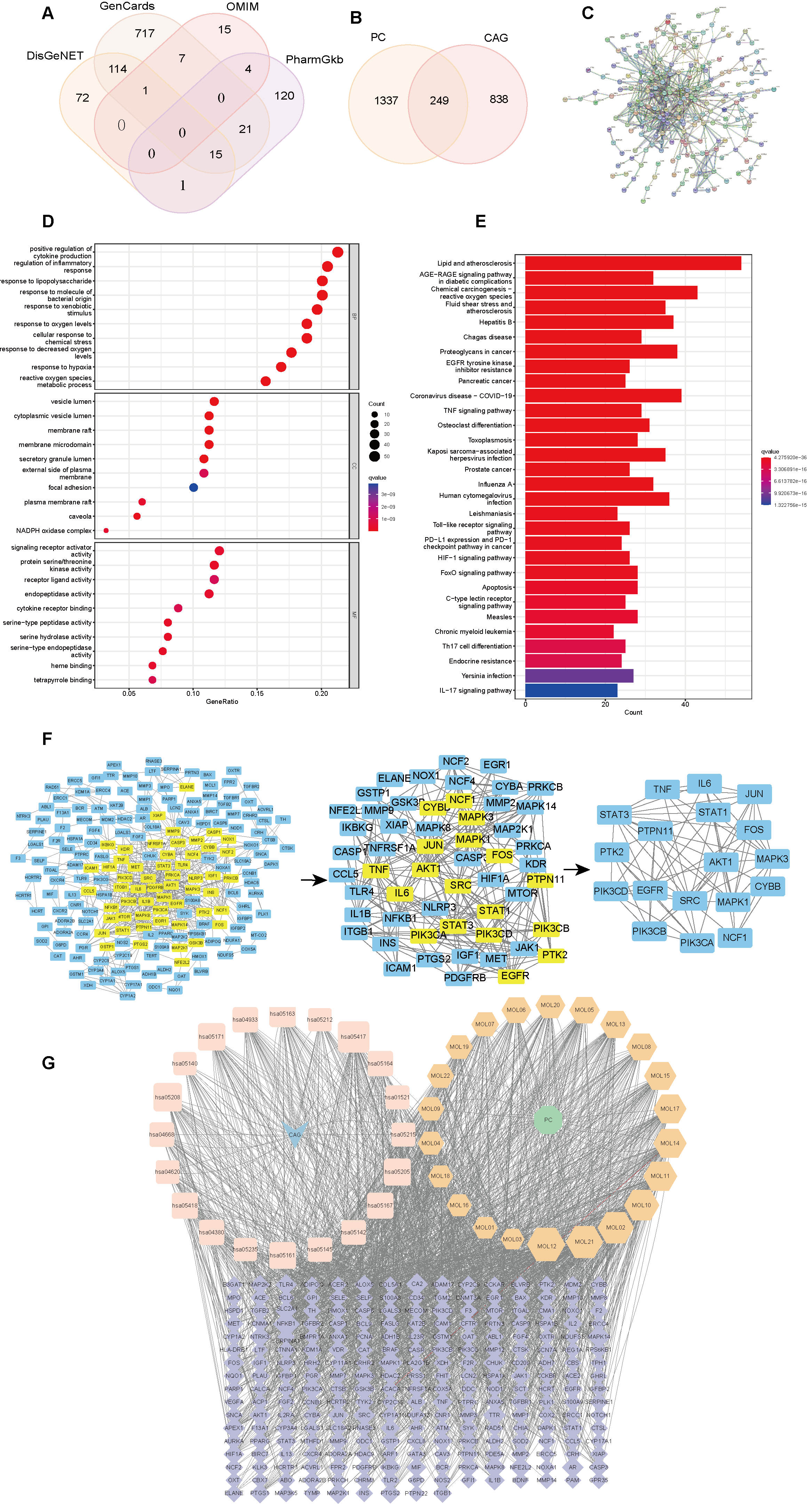

Supplement: Supplementary file 1 [file pharmaceuticals-18-00537-s001.zip › Supplementary Figure S1 Network pharmacological analysis .tif]
